# Anxiolytic and Hypnotic Effects of Aqueous and Ethanolic Extracts of Aerial Parts of Echium italicum L. in Mice

**Published:** 2012-05-28

**Authors:** Hossein Hosseinzadeh, Shabnam Shahandeh, Shabnam Shahsavand

**Affiliations:** 1 Department of Pharmacodynamy and Toxicology, School of Pharmacy, Mashhad University of Medical Sciences, Mashhad, IR Iran

**Keywords:** Hypnotics and Sedatives, Rotarod Performance Test, Anti-Anxiety Agents

## Abstract

**Background:**

Research in the area of herbal psychopharmacology has clearly improved in recent decades. Self-administration of herbal medicines has been the most popular therapeutic alternative to standard medicine.

**Objectives:**

Since the extract of *Echium amoenum* exhibits an anxiolytic effect, the aim of this study is to evaluate the anxiolytic and hypnotic effects in mice of the aqueous and ethanolic extracts of aerial parts of *E. italicum*, a member of the Boraginaceae family.

**Materials and Methods:**

Mice were administered the agents intraperitoneally before the start of the experiments for evaluation of hypnotic activity (induced by sodium pentobarbital, 30 mg/kg, i.p.), anxiolytic activity (elevated plus-maze [EPM] test), locomotor activity (open field test), and motor coordination (rotarod test).

**Result:**

The ethanolic and aqueous extracts of *E. italicum*, at doses of 1.2 and 2.1 g/kg, increased the percentage of time-spent and the percentage of arm entries in the open arms of the EPM and decreased the percentage of time-spent in the closed arms of the EPM. Moreover, both extracts decreased the pentobarbital-induced latency to sleep and significantly increased the total sleeping time induced by pentobarbital. In addition, locomotor activity was affected by aqueous extracts and ethanolic extract (at higher doses). Both extracts showed no effect in the rotarod test.

**Conclusions:**

These results suggest that both ethanolic and aqueous extracts of *E. italicum* may have anxiolytic effects and sedative activity but no effect on muscle relaxation.

## 1. Background

Research into herbal psychopharmacology has clearly improved in recent decades. This improvement may be due to the fact that self-administration of herbal medicines has been the most popular therapeutic alternative to standard medicine. The use of herbal medications by physicians in Europe and Asia is becoming very common, and researchers are exploring traditional remedies to find suitable cures for mind-altering diseases. Anxiety disorders are the most common mental diseases in the world and are an important area of research in psychopharmacology. Interest in alternative medicine and plant-derived medications that affect the mind is growing. *Echium italicum L*. (Italian bugloss) is an important medicinal plant belonging to the Boraginaceae family. It is naturally distributed throughout Australia, Europe, the Mediterranean region, and Asia, including Iran and neighboring areas (1). Red colored root preparations of the plant have long been used as a remedy, especially to treat burns and wounds. Previous phytochemical studies revealed that the roots of at least 150 species of the Boraginaceae family contain naphthoquinone pigments such as alkannin and shikonin derivatives, which are two enantiomeric dyes extracted from *Alkanna tinctoria* and *Lithospermum erythrorhizon*, respectively (2-5). Alkannin (S-enantiomer), shikonin (R-enantiomer) and related derivatives are potent pharmaceutical substances with a well-established and broad spectrum of properties, such as wound healing (2, 3, 6), antibacterial (7), anti-HIV (8), anti-tumor (9, 10), anti-inflammatory (11-13) and antioxidant activities (14). In addition, these compounds have been used as colorants in cosmetics and food industries (15). A home-made ointment prepared from pulverized roots of *E. italicum L*. (EI) and lard, traditionally used in Macedonian medicine, has long been known to exhibit strong anti-inflammatory and wound healing effects, and these reddish roots may contain derivatives of either shikonin or alkannin, enantiomers with similar chemical and biological properties (4).

*E. amoenum* also belongs to the Boraginaceae family and is a biennial herb indigenous to a narrow zone of northern Iran and the Caucasus where it grows at an altitude up to 2200 m (1). The dried decoction of the violet-blue petals of *E. amoenum* has long been known to have tranquillizing effects among the Iranian people (16). One report suggests that the extract of *E. amoenum* seems to possess anxiolytic effects with lower sedative activity than that of diazepam (17).

## 2. Objectives

The aim of this study was to evaluate the anxiolytic and hypnotic effects in mice of different doses of aqueous and ethanolic extracts of aerial parts of *E. italicum*, another member of the Boraginaceae family.

## 3. Materials and Methods

### 3.1. Animals

Male BALB/c mice, weighing between 25-30 g, were obtained from the Razi Serum Organization and maintained at 21±2°C on a 12h/12h light cycle in the animal room of the Mashhad School of Pharmacy. Food pellets and tap water were available ad libitum and each mouse was used once for experimentation.

### 3.2. Plants

Aerial parts of *E. italicum* were collected from the Tandooreh Mountains at a height of 1600 m in Dargaz (Razavi Khorasan province, Iran). The plant was air dried in the shade (20-25 °C) and sent to the Mashhad School of Pharmacy. The plant was then identified by the herbarium at the School of Pharmacy, Mashhad, and a sample were deposited under the code number 040-0209-06.

### 3.3. Extraction

Aqueous extract: The dried aerial parts (200 g) were powdered and boiled in 2 liters of water for 15 minutes. After filtration, the extract-containing plates were heated at 40°C until the solvent evaporated. The brown leftover extract was kept in a refrigerator until its administration. Normal saline with a few drops of Tween 80 was used as a solvent. The yield was 9.195%. Ethanolic extract: First, petroleum ether was used to remove the plant’s fat content. Ethanol (95%) was then used to macerate the dried plant for 48 hours. After filtration, the solvent was evaporated at 30°C. The leftover extract was kept in the refrigerator until its administration. Normal saline with a few drops of Tween 80 was used as a solvent. The yield was 3.39%.

### 3.4. Acute Toxicity Study

To determine the lethal dose 50% (LD_50_) and maximum tolerated dose, doses of 3.5 g to 5 g of each extract were injected intraperitoneally (IP) into mice. Five mice were used for each dose group, and mortality was recorded after 24 hours.

### 3.5. Preliminary Phytochemical Tests

Phytochemical screening of aqueous and ethanolic extracts for alkaloids, flavonoids, saponins and tannins was performed according to the literature (18).

### 3.6. Agent Administration

The following agents were administered IP to groups of 8 mice, 30 minutes before the test:

- Negative control: Normal saline 10 ml/kg for aqueous extract and normal saline with Tween 80 for ethanolic extract.

- Positive control: Diazepam 3 mg/kg, pentobarbital 30 mg/kg.

- Aqueous extract and alcoholic extract: 0.3, 0.6, 1.2, 2.1 g/ kg of *E. italicum*.

### 3.7. Rotarod Test

A rotarod test was performed 30 and 60 minutes after administration of extracts, based on previously described methods. All mice were given 1 trial in the morning the day before the test and again in the afternoon. The mice with the best scores (longest latency on the rotarod) were retained for definitive testing. These mice were randomly divided into 10 groups. On the study day and approximately 30 and 60 min after extract or vehicle control administration, the mice were tested in the rotarod study. The mice were placed on the rod (3 cm diameter), which rotated at an initial rate of 10 rpm and then at a constant rate of 20 rpm, rotating in the direction opposite to the animals. The time elapsed up to 300 s or until the mice fell was recorded (19).

### 3.8. Open-Field Test

The open field test was planned to evaluate behavioral responses such as locomotor activity, hyperactivity and exploratory behaviors (20, 21). This test was performed because mouse mobility affects their ability to move or climb. In this test, the effects of administration on the animals’ mobility were calculated. Each mouse was placed in the center of the open field apparatus, and its behavior was observed for 5 min. The parameters evaluated were the total number of squares crossed, the number of outer squares (those adjacent to the walls) crossed, and the number of inner squares crossed. These three measures are referred to as total (TL), peripheral (PL), and central locomotion (CL), respectively. The numbers of leanings (one or two paws in contact with the wall), rearings (the mouse standing on its two hind paws without touching the walls), groomings (face cleaning, paw licking, fur licking, head scraping, and rubbing), and defecations were also recorded. At the end of each test, the whole area was cleaned with a wet sponge and a dry paper towel.

### 3.9. Elevated Plus-Maze

The EPM consisted of two open arms and two closed arms (10 cm wide × 50 cm long) perpendicular to each other and elevated 50 cm from the floor. The walls of the closed arms were 40 cm high. The arms were connected by a central square measuring 10 cm × 10 cm. The animal was placed in the center of the apparatus facing an open arm, and its behavior was recorded for 5 min. The maze was situated in a dimly lit room and, to minimize olfactory cues between trials, and was cleaned between animals. The following variables were recorded: number of open and closed arm entries and time spent in the open and closed arms. The percentage of time spent in the open arms (% OAT) and the percentage of open arms entries (% OAE) were used as anxiety measures. The number of open arms plus closed arms entries was used as a measure of activity. An entry was counted whenever the animal crossed with all four paws into an arm. Increased % OAT or % OAE is indicative of a reduced anxiety state in the EPM (22).

### 3.10. Prolongation Effect on Pentobarbital-Induced Sleeping Time

Test samples suspended in normal saline were administered to the animals. All experiments were carried out between 1 pm and 5 pm. Animals were denied food for 24 h prior to the experiment. Thirty minutes after the intraperitoneal administration (i.p.) of test samples, pentobarbital was i.p. administered at a dose of 30 mg/kg body weight to mice placed in a box. The animals in the box that stopped moving within 15 min after pentobarbital injection were immediately transferred to another box, whereas those that remained immobile for more than 3 min were judged to be asleep. The sleeping time was defined as the time from their transfer into the second box to when they resumed spontaneous movements. Mice that failed to fall asleep within 15 min after pentobarbital administration were excluded from the experiments (23).

### 3.11. Statistical Analysis

PCS software was used for LD_50_ calculation. Data are expressed as mean ± standard error of the mean (SEM). Statistical analysis was performed using one-way ANOVA followed by the Tukey-Kramer *post-hoc* test for multiple comparisons. *P*-values less than 0.05 were considered statistically significant.

## 4. Results

### 4.1. Preliminary Phytochemical Tests

Preliminary phytochemical tests indicated that the aqueous and ethanolic extracts were rich in flavonoids and tannins. The extract did not contain alkaloids and saponins.

### 4.2. Maximum Tolerated Dose and Acute Toxicity

The LD_50_ and maximum tolerated dose (MTD) values of the aqueous extract were 4 and 3 g/kg, respectively. The ethanolic extract showed no mortality up to a dose of 5g/ kg.

### 4.3. Effect of E. italicum Extracts on the Elevated Plusmaze

**Figure 1:**
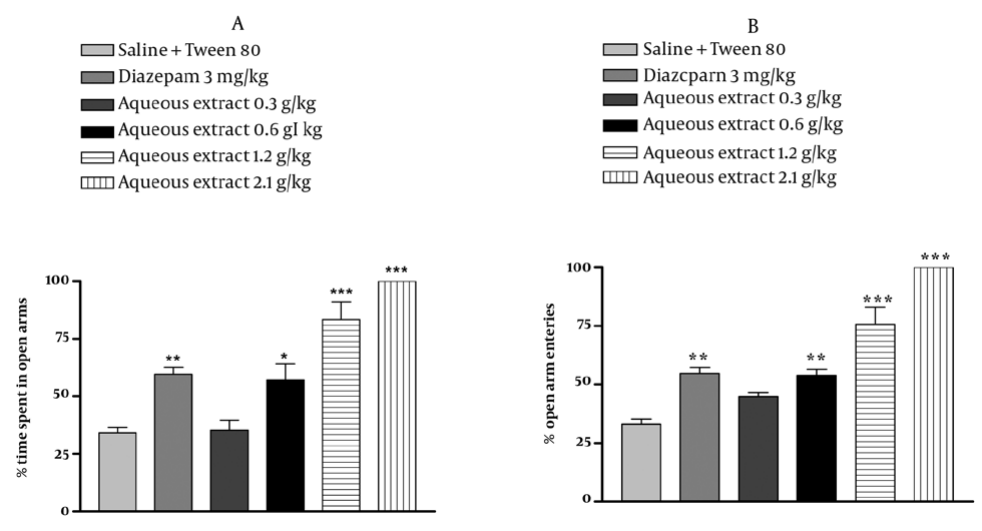
Effects of E. italicum aqueous extract on (a) the percentage of time spent in open arms and (b) the percentage of open arm entries of the elevated plus-maze (EPM). Data are presented as mean values ±S.E.M. from a group of six mice. *, ** and *** indicate P < 0.05, P < 0.01 and P < 0.001, respectively. Compared with normal saline + Tween 80-treated control. Tukey-Kramer test.

**Figure 2:**
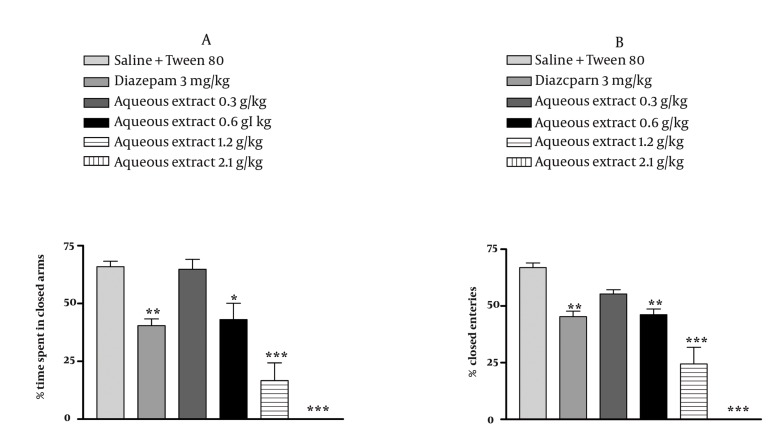
Effects of E. italicum aqueous extract on (a) the percentage of time spent in the closed arms and (b) the percentage of closed arm entries of the elevated plus-maze (EPM). Data are presented as mean values ± S.E.M. from a group of six mice. *, ** and *** indicate P < 0.05, P < 0.01 and P < 0.001, respectively. Compared with normal saline + Tween 80-treated control. Tukey-Kramer test.

**Figure 3:**
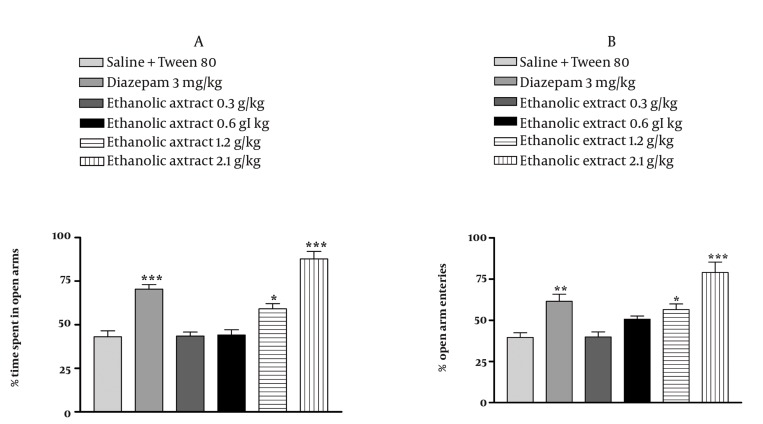
Effects of E. italicum ethanolic extract on (a) the percentage of time spent in open arms and (b) the percentage of open arm entries of the elevated plus-maze (EPM). Data are presented as mean values ±S.E.M. from a group of six mice. *, ** and *** indicate P < 0.05, P < 0.01 and P < 0.001, respectively. Compared with normal saline + Tween 80-treated control. Tukey-Kramer test.

**Figure 4:**
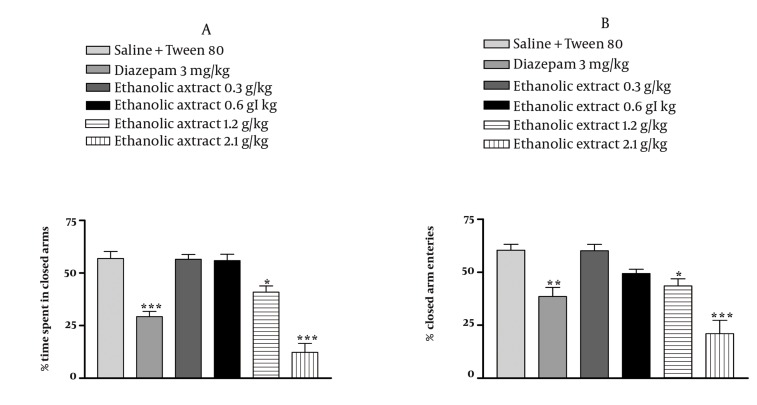
Effects of E. italicum ethanolic extract on (a) the percentage of time spent in closed arms and (b) the percentage of closed arm entries of the elevated plus-maze (EPM). Data are presented as mean values ±S.E.M. from a group of six mice. *, ** and *** indicate P < 0.05, P < 0.01 and P < 0.001, respectively. Compared with normal saline + Tween 80-treated control. Tukey-Kramer test.

In the elevated plus-maze, the behavior observed confirmed the anxiolytic activity of diazepam as reported previously. The aqueous extract of *E. italicum* at doses at 0.6, 1.2 and 2.1 g/kg increased the percentage of time spent and the percentage of arm entries in the open arms (*P* < 0.05 *P* < 0.001 and *P* < 0.001, respectively) (Figure 1a, b) and decreased the percentage of time spent and percentage of arm entries in the closed arms (*P* < 0.05, *P* < 0.001 and *P* < 0.001, respectively) (Figure 2a, b). The ethanolic extract of *E. italicum* at doses of 1.2 and 2.1 g/kg increased the percentage of time spent and percentage of arm entries in the open arms (*P* < 0.05 and *P* < 0.001, respectively) (Figure 3a, b) and decreased the percentage of time spent and percentage of arm entries in the closed arms (*P* < 0.05 and *P* < 0.001, respectively) (Figure 4a, b).

### 4.4. Effects of E. italicum Extract on Pentobarbital-Induced Sleeping Time

**Figure 5:**
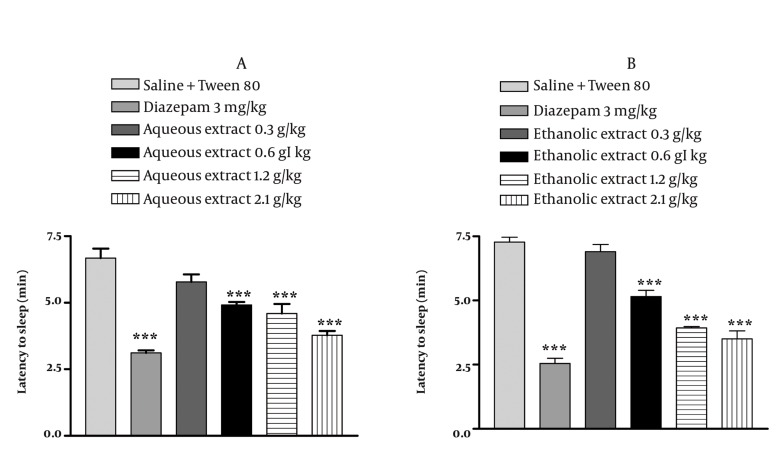
Effects of E. italicum extracts on the latency to loss of the righting reflex. After 30 min pretreatment with the plant extracts or vehicle, animals were injected with pentobarbital (30 mg/kg, i.p.). The interval between the administrations of pentobarbital until the loss of the righting reflex was recorded as onset of sleep. Results are represented as means ± S.E.M. from six mice. *, ** and *** indicate P < 0.05, P < 0.01 and P < 0.001, respectively. Tukey-Kramer test.

**Figure 6:**
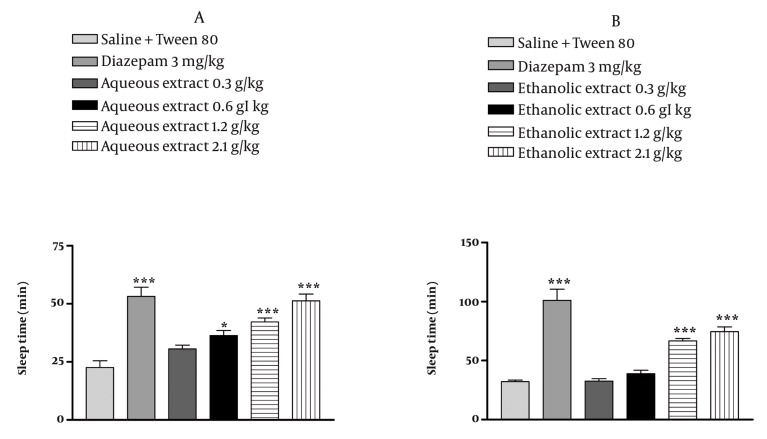
Effects of E. italicum extracts on the time with loss of righting reflex. After 30 min pretreatment with the plant extracts or vehicle, animals were injected with pentobarbital (30 mg/kg, i.p.). The time spent with loss of the righting reflex was recorded as sleep time. Results are represented as means ± S.E.M. from six mice. *, ** and *** indicate P < 0.05, P < 0.01 and P < 0.001, respectively. Tukey-Kramer test.

Results are reported in Figures 5 and 6. In saline treated animals, the righting reflex was lost after 6.68 min of pentobarbital injection. The injection of *E. italicum* aqueous extract at doses of 0.6, 1.2 and 2.1 g/kg and diazepam significantly suppressed latency to sleep by 26%, 41%, 43% and 53.43%, respectively (*P* < 0.001) (Figure 5a). *E. italicum* ethanolic extract at doses of 0.6, 1.2 and 2.1 g/kg and diazepam significantly suppressed latency to sleep by 29%, 45%, 51% and 65%, respectively (*P* < 0.001) (Figure 5b). The total duration of sleep was affected significantly by *E. italicum* aqueous extract at doses of 1.2 and 2.1 g/kg and diazepam by 86%, 126% and 135%, respectively (*P* < 0.001) (Figure 6a), and similar effects were caused by *E. italicum* ethanolic extract (*P* < 0.001) (Figure 6b).

### 4.5. Effect of E. italicum Extracts on Motor Activity in the Open Field Test

**Figure 7:**
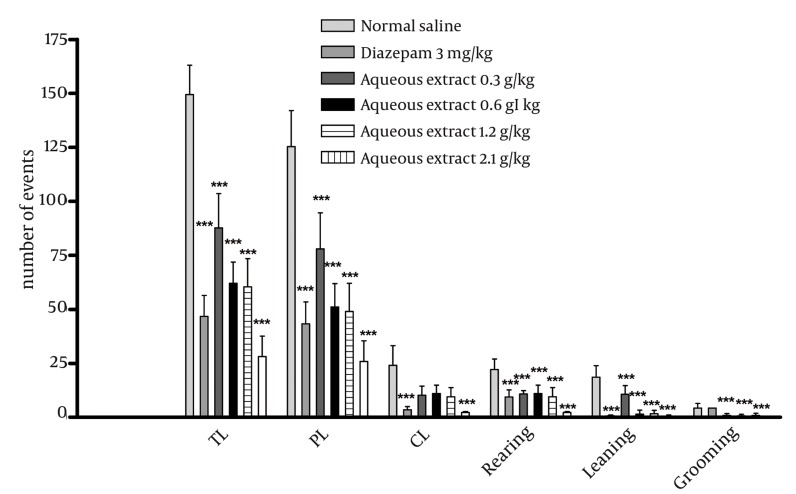
Effects of the aqueous extract in the open-field test. Results are shown as mean ± SEM (n=6). Different doses of aqueous extract are compared to normal saline + Tween 80. *, ** and *** indicate P < 0.05, P < 0.01 and P < 0.001, respectively. Tukey-Kramer test. Abbreviations: CL: Central Locomotion; TL: Total Locomotion; PL: Peripheral Locomotion

Aqueous extract (Figure 7) caused a significant decrease in all motor activity factors (total [TL], peripheral [PL] and central locomotion [CL], and the number of leanings, rearings, groomings and defecations [*P* < 0.001]) at all doses. Ethanolic extract at doses of 1.2 and 2.1 g/kg decreased the number of CL, TL and leanings (*P* < 0.001). Ethanolic extract at doses of 0.6, 1.2 and 2.1 g/kg also decreased the number of rearings (*P* < 0.001).

### 4.6. Effect of E. italicum Extract on Motor Coordination

No alteration was observed in the rotarod test 30 and 60 min after treatment with aqueous and ethanolic extracts (0.3, 0.6, 1.2 and 2.1 g/kg) compared with normal saline, while diazepam (3 mg/kg) in a muscle relaxant dose decreased this parameter by 83%, as expected.

## 5. Discussion

The results showed that the aqueous extract at doses of 0.6, 1.2 and 2.1 g/kg, and ethanolic extract at doses of 1.2 and 2.1 g/kg, increased the percentage of open arm entries and the time spent in them, decreased latency to sleep and increased total sleep time. In the open field test, all doses of aqueous extract decreased the number of PL, CL, TL, groomings, leanings, rearings and defecations. The ethanolic extract at doses of 1.2 and 2.1 g/kg decreased the number of CL, TL and leanings. The ethanolic extract at doses of 0.6, 1.2 and 2.1 g/kg also decreased the number of rearings. Neither extract had any effect on motor coordination.

Anxiety disorders, such as generalized anxiety disorder (GAD), social phobia and post-traumatic stress disorder, present with marked psychological anxiety and distress (24). The pathophysiology of anxiety disorders is still unknown, although recent evidence indicates that neurobiological abnormalities in serotonergic, noradrenergic, glutamatergic and GABAergic transmission may be involved (25). The contribution of these pathways is reflected in the efficacy of selective serotonin reuptake inhibitors (SSRIs), selective serotonin and noradrenalin reuptake inhibitors (SNRIs) and benzodiazepines (26). Phytotherapeutic interventions, such as the use of *P. methysticum*, which may help in anxiety disorders, are classed as anxiolytics and generally have effects on the GABA system (27). Other mechanisms that may be involved include ionic channel transmission by blockage of voltage gates, blockage or alteration of membrane structures, (28) GABA transaminase or glutamic acid decarboxylase inhibition (29), or less commonly via binding with benzodiazepine receptor sites (e.g., the a subunit) (30). Subsequent increased GABA neurotransmission has a damping effect on stimulatory pathways, which ultimately provides a psychologically calming effect (31). A novel study by Awad and colleagues (29) was conducted to determine whether several common botanical products directly affect the primary brain enzymes responsible for GABA metabolism. In vitro rat brain homogenate assays revealed that the aqueous extract of *Melissa officinalis* (lemon balm) exhibited the greatest inhibition of GABA transaminase activity among the preparations assessed. Matricaria recutita (chamomile) and *Humulus lupulus* (hops), meanwhile, showed significant inhibition of glutamic acid decarboxylase activity.

Flavonoids are natural active compounds that tend to bind to benzodiazepine GABA_A_ receptors, and they act pharmacologically as partial agonists. Some semi-synthetic flavone derivatives are much more potent than diazepam in vivo (32-34). Baicalin, a flavonoid extracted from *Scutellaria lateriflora L*., exerts anxiolytic activity that is antagonized by a GABA_A_-specific antagonist (35). It has also been suggested that chyrsin, another natural flavonoid from *Passiflora coerulea*, exerts anxiolytic effects without any sedative and muscle relaxation activities. Chyrsin can decrease flurazepam binding to its binding site on the BDZ receptor, and its anxiolytic effect is antagonized by flumazenil, a GABA_A_ antagonist. It can therefore be a partial agonist of the GABA_A_ receptor (36). Wogonin is a flavonoid from *Scutellaria baicalensis Georgi* that acts in a similar manner (37). Flavonoid effects are probably also related to the anxiolytic effect of *E. italicum* extracts. Tannins cannot enter the central nervous system because of their polar structure and therefore are not involved in these effects.

It has been reported that almost all members of the Boraginaceae family have pyrrolizidine alkaloids, compounds that lead to sinusoidal-obstruction syndrome (38). These alkaloids may also be mutagenic and hepatocarcinogenic (39, 40) and the hepatotoxicity of some *Echium* species is related to these alkaloids (41). Based on phytochemical tests, both ethanolic and aqueous extracts have tannins and flavonoids but no alkaloids, and they have higher LD_50_ values than other members of the Boraginaceae family. The ethanolic extract was even less toxic. The LD*50* and MTD values of the aqueous extract were 4 and 3 g/kg, respectively. The ethanolic extract showed no mortality up to a dose of 5 g/kg (42). Our research shows that both extracts have anxiolytic and hypnotic effects, and the aqueous extract was more effective than the ethanolic extract. Neither extract had any effect on motor coordination.
